# Bis(2,2′-bipyridine-κ^2^ *N*,*N*′)bis­(1*H*-indole-2-carboxyl­ato-κ^2^ *O*,*O*′)cadmium–2,2′-bipyridine–water (1/0.5/2)

**DOI:** 10.1107/S1600536811021064

**Published:** 2011-06-11

**Authors:** Yun-Xia Li, Bi-Song Zhang, Miao Zheng

**Affiliations:** a College of Pharmaceutics and Material Engineering, Jinhua College of Profession and Technology, Jinhua, Zhejiang 321007, People’s Republic of China

## Abstract

The asymmetric unit of title compound, [Cd(C_9_H_6_NO_2_)_2_(C_10_H_8_N_2_)_2_]·0.5C_10_H_8_N_2_·2H_2_O, consists of one complex mol­ecule, one half of an uncoordinated 2,2′-bipyridine mol­ecule and two solvent water mol­ecules. The uncoordinated 2,2′-bipyridine mol­ecule is located on a center of symmetry. Within the complex mol­ecule, the Cd^II^ atom is coordinated by four N atoms from two 2,2′-bipyridine ligands and three O atoms from two 1*H*-indole-2-carboxyl­ate anion ligands, completing a distorted CdN_4_O_3_ penta­gonal bipyra­mid. The mol­ecules are assembled into one-dimensional chains along the [100] direction through classical hydrogen bonds (O—H⋯N, N—H⋯O and O—H⋯O). The resulting chains are further connected into two-dimensional supra­molecular layers parallel to the (110) direction by inter­molecular classical hydrogen bonds (N—H⋯O and O—H⋯O) from adjacent chains. A three-dimensional supra­molecular network is formed *via* interlayer and O—H⋯O hydrogen bonds.

## Related literature

For general background, see: Dillon *et al.* (2003 ▶). For related cadmium(II) complexes with bipyridine and 1,10-phenanthroline ligands, see: Zhang *et al.* (2005 ▶); Lou & Zhang (2007 ▶).
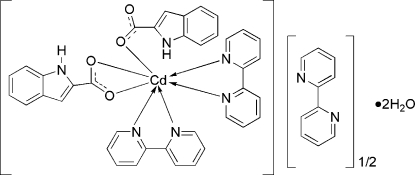


## Experimental

### 

#### Crystal data

[Cd(C_9_H_6_NO_2_)_2_(C_10_H_8_N_2_)_2_]·0.5C_10_H_8_N_2_·2H_2_O
*M* *_r_* = 859.19
Triclinic, 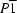

*a* = 11.513 (2) Å
*b* = 12.945 (3) Å
*c* = 14.302 (3) Å
α = 114.95 (3)°
β = 94.27 (3)°
γ = 93.84 (3)°
*V* = 1915.8 (9) Å^3^
*Z* = 2
Mo *K*α radiation
μ = 0.63 mm^−1^
*T* = 290 K
0.26 × 0.19 × 0.06 mm

#### Data collection

Rigaku R-AXIS RAPID diffractometer
Absorption correction: multi-scan (*ABSCOR*; Higashi, 1995 ▶) *T*_min_ = 0.865, *T*_max_ = 0.963
15180 measured reflections
6724 independent reflections
4444 reflections with *I* > 2σ(*I*)
*R*_int_ = 0.058

#### Refinement

*R*[*F* ^2^ > 2σ(*F* ^2^)] = 0.044
*wR*(*F* ^2^) = 0.106
*S* = 1.17
6724 reflections
515 parameters
H-atom parameters constrained
Δρ_max_ = 1.26 e Å^−3^
Δρ_min_ = −1.55 e Å^−3^

### 

Data collection: *RAPID-AUTO* (Rigaku, 1998 ▶); cell refinement: *RAPID-AUTO*; data reduction: *CrystalStructure* (Rigaku/MSC, 2004 ▶); program(s) used to solve structure: *SHELXS97* (Sheldrick, 2008 ▶); program(s) used to refine structure: *SHELXL97* (Sheldrick, 2008 ▶); molecular graphics: *ORTEPII* (Johnson, 1976 ▶) and *DIAMOND* (Brandenburg & Putz, 2004 ▶); software used to prepare material for publication: *SHELXL97*.

## Supplementary Material

Crystal structure: contains datablock(s) global, I. DOI: 10.1107/S1600536811021064/rk2274sup1.cif

Structure factors: contains datablock(s) I. DOI: 10.1107/S1600536811021064/rk2274Isup2.hkl

Additional supplementary materials:  crystallographic information; 3D view; checkCIF report

## Figures and Tables

**Table 1:** Hydrogen-bond geometry (Å, °)

*D*—H⋯*A*	*D*—H	H⋯*A*	*D*⋯*A*	*D*—H⋯*A*
N5—H5*C*⋯O5^i^	0.86	2.09	2.892 (9)	155
N6—H6*C*⋯O2^ii^	0.86	2.00	2.801 (8)	156
O5—H5*A*⋯O6	0.82	1.96	2.748 (8)	161
O5—H5*B*⋯N7^iii^	0.82	2.09	2.879 (8)	161
O6—H6*A*⋯O5^iv^	0.82	2.01	2.781 (7)	156
O6—H6*B*⋯O3^v^	0.82	1.95	2.778 (6)	174

Symmetry codes: (i) 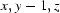; (ii) 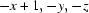; (iii) 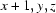; (iv) 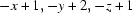; (v) 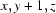.

**Fig. 1.**
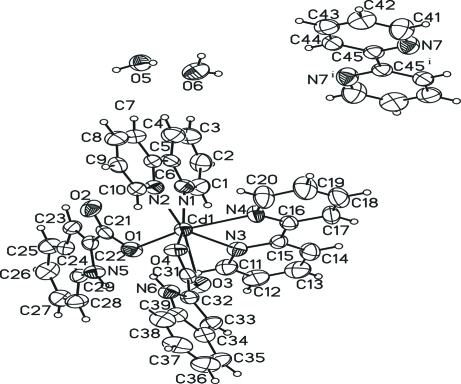
The molecule structure of the title compound, showing the atom-labelling scheme. Displacement ellipsoids are drawn at the 50% probability level. H atoms are presented as a small spheres of arbitrary radius. Symmetry code: (i) -x, -y + 2, -z + 1.

**Fig. 2.**
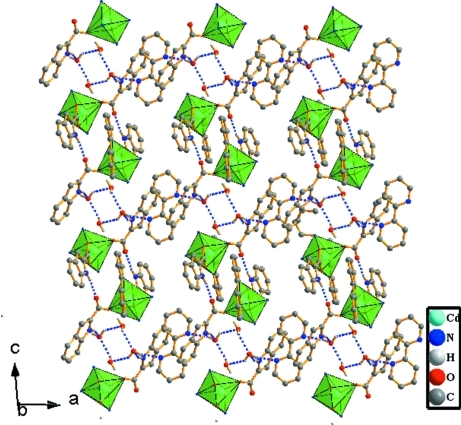
A packing diagram of the title compound. H atoms are omitted for clarity.

## supplementary crystallographic information

### Comment

The indole-2-carboxylic acid moiety is present in a great number of molecules with a broad spectrum of pharmacological activity, anticonvulsant, antihypertensive and antifungal properties. It has been found that the biological activities of the therapeutic agents are considerably increased when they are bonded into metal complex molecules (Dillon *et al.*, 2003). Cadmium(II) ion with 1.10-phenanthroline and pyridine ligands can respectively form CdN_4_O_4_ dodecahedron and CdN_4_O_3_ polyhedron complex molecules (Lou & Zhang, 2007, Zhang *et al.*, 2005). In this paper, we report synthesis and structure of a new cadium coordination complex with indole-2-formic acid and 2,2'-bipyridine ligands. The crystal structure of the title compound consists of one complex Cd(C_10_H_8_N_2_)_2_(C_9_H_7_NO_2_)_2_ molecule, one half uncoordinated 2,2'-bipyridine molecule and two lattice water molecules (Fig. 1). The uncoordinated 2,2'-bipyridine molecule placed in the center of symmetry (0, 1, 1/2 ). In the complex molecule, the Cd^II^ atom is coordinated by four N atoms from two 2,2'-bipyridine ligands, three O atoms from two 2-indolyl-formic acid anions ligands, completing a distorted CdN_4_O_3_ pentagonal bipyramid. The equatorial positions of the Cd^II^ ion are occupied by two carboxylate O atoms (O3, O4) and three N atoms (N2, N1, N3) from different 2,2'-bipyridine molecules, and the axial ones by the other one N atom (N4) and one carboxylate O atom (O1). The Cd1—N bond length is 2.364 (4) Å to 2.450 (5) Å, and Cd1—O bond lengths are 2.239 (4) Å and 2.653 (4) Å. In the crystal structure, classical O5—H5B···N6, N6—H6C···O2^ii^ and O6—H6A···O5^iv^ hydrogen bonding interactions (Table 1) form one-dimensional chains. The chains are connected with each other *via* intermolecular hydrogen bonds (N5—H5C···O5^i^, O6—H6A···O5^iv^) from adjacent chains to form a two-dimensional layer (Fig. 2). Furthermore, a three-dimensional network is formed *via* intermolecular forces between layers and hydrogen bonds (O6—H6A···O5^iv^). Symmetry codes: (i) *x*, *y* - 1, *z*; (ii) -*x* + 1, -*y*, -*z*; (iv) -*x* + 1, -*y* + 2, -*z* + 1.

### Experimental

CdCl_2_.6H_2_O (0.11 g, 0.54 mmol) was dissolved in appropriate amount of water, and then 1*M* Na_2_CO_3_ solution was added. CdCO_3_ was obtained by filtration, which was then washed with distilled water for 5 times. The freshly prepared CdCO_3_, 2-indolyl-formic acid (0.0725 g, 0.5 mmol), 2,2'-bipyridine (0.0781 g, 0.5 mmol), CH_3_OH/H_2_O (*v*/*v* = 1:2, 15 ml) were mixed and stirred for 2.0 h. Subsequently, the resulting cream suspension was heated in a 23 ml Teflon-lined stainless steel autoclave at 433 K for 5800 min. After that, autoclave was cooled to room temperature according to the procedure at 2600 min. The solid was filtered off. The resulting filtrate was allowed to stand at room temperature, and slow evaporation for 4 months afforded colourless block single crystals.

### Refinement

The C-bound H atoms were placed in calculated positions, with C—H = 0.93 Å and *U*_iso_(H) = 1.2*U*_eq_(C), and were refined using the riding-model approximation. The H atoms of the water molecule were located in a difference Fourier map and refined with an O—H distance restraint of 0.82 Å and *U*_iso_(H) = 1.5*U*_eq_(O). H atoms attached to N atoms were placed in calculated positions, with N—H = 0.86 Å and *U*_iso_(H) = 1.2*U*_eq_(N), and were refined using a riding model. The largest peak in the final difference Fourier map is 1.49 Å from atom Cd1 and the deepest hole is 0.94 Å from atom Cd1.

### Crystal data

**Table:**

[Cd(C_9_H_6_NO_2_)_2_(C_10_H_8_N_2_)_2_]·0.5C_10_H_8_N_2_·2H_2_O	*Z* = 2
*M**_r_* = 859.19	*F*(000) = 878
Triclinic, *P*1	*D*_x_ = 1.489 Mg m^−^^3^
Hall symbol: -P 1	Mo *K*α radiation, λ = 0.71073 Å
*a* = 11.513 (2) Å	Cell parameters from 10384 reflections
*b* = 12.945 (3) Å	θ = 3.2–25°
*c* = 14.302 (3) Å	µ = 0.63 mm^−^^1^
α = 114.95 (3)°	*T* = 290 K
β = 94.27 (3)°	Block, colourless
γ = 93.84 (3)°	0.26 × 0.19 × 0.06 mm
*V* = 1915.8 (9) Å^3^	

### Data collection

**Table:**

Rigaku R-AXIS RAPID diffractometer	6724 independent reflections
Radiation source: fine-focus sealed tube	4444 reflections with *I* > 2σ(*I*)
graphite	*R*_int_ = 0.058
ω scans	θ_max_ = 25.0°, θ_min_ = 3.1°
Absorption correction: multi-scan (*ABSCOR*; Higashi, 1995)	*h* = −13→12
*T*_min_ = 0.865, *T*_max_ = 0.963	*k* = −15→15
15180 measured reflections	*l* = −16→16

### Refinement

**Table:**

Refinement on *F*^2^	Secondary atom site location: difference Fourier map
Least-squares matrix: full	Hydrogen site location: inferred from neighbouring sites
*R*[*F*^2^ > 2σ(*F*^2^)] = 0.044	H-atom parameters constrained
*wR*(*F*^2^) = 0.106	*w* = 1/[σ^2^(*F*_o_^2^) + (0.*P*)^2^ + 4.4876*P*] where *P* = (*F*_o_^2^ + 2*F*_c_^2^)/3
*S* = 1.17	(Δ/σ)_max_ < 0.001
6724 reflections	Δρ_max_ = 1.26 e Å^−^^3^
515 parameters	Δρ_min_ = −1.55 e Å^−^^3^
0 restraints	Extinction correction: *SHELXL97* (Sheldrick, 2008), Fc^*^=kFc[1+0.001xFc^2^λ^3^/sin(2θ)]^-1/4^
Primary atom site location: structure-invariant direct methods	Extinction coefficient: 0.0015 (2)

### Special details

**Table:**

Geometry. All s.u.'s (except the s.u. in the dihedral angle between two l.s. planes) are estimated using the full covariance matrix. The cell s.u.'s are taken into account individually in the estimation of s.u.'s in distances, angles and torsion angles; correlations between s.u.'s in cell parameters are only used when they are defined by crystal symmetry. An approximate (isotropic) treatment of cell s.u.'s is used for estimating s.u.'s involving l.s. planes.
Refinement. Refinement of *F*^2^ against ALL reflections. The weighted *R*-factor *wR* and goodness of fit *S* are based on *F*^2^, conventional *R*-factors *R* are based on *F*, with *F* set to zero for negative *F*^2^. The threshold expression of *F*^2^ > σ(*F*^2^) is used only for calculating *R*-factors(gt) *etc*. and is not relevant to the choice of reflections for refinement. *R*-factors based on *F*^2^ are statistically about twice as large as those based on *F*, and *R*-factors based on ALL data will be even larger.

### Fractional atomic coordinates and isotropic or equivalent isotropic displacement parameters (Å^2^)

**Table:**

	*x*	*y*	*z*	*U*_iso_*/*U*_eq_	
Cd1	0.36632 (4)	0.14575 (4)	0.19144 (3)	0.03937 (15)	
N1	0.3953 (4)	0.3521 (4)	0.2404 (4)	0.0463 (12)	
N2	0.3905 (4)	0.1847 (4)	0.0463 (3)	0.0414 (11)	
N3	0.2855 (4)	0.2010 (4)	0.3542 (3)	0.0421 (11)	
N4	0.1600 (4)	0.1689 (4)	0.1760 (4)	0.0512 (13)	
N5	0.7181 (4)	0.2314 (4)	0.4379 (3)	0.0427 (11)	
H5C	0.6805	0.1670	0.4272	0.051*	
N6	0.2909 (4)	−0.2837 (4)	−0.0315 (3)	0.0460 (12)	
H6C	0.3187	−0.2581	−0.0727	0.055*	
N7	−0.1248 (4)	0.9667 (5)	0.4058 (4)	0.0660 (15)	
O1	0.5438 (3)	0.1593 (3)	0.2739 (3)	0.0506 (10)	
O2	0.6372 (3)	0.2675 (4)	0.2080 (3)	0.0620 (12)	
O3	0.3234 (4)	−0.0430 (3)	0.2186 (3)	0.0583 (11)	
O4	0.3252 (3)	−0.0426 (3)	0.0641 (3)	0.0558 (11)	
O5	0.6427 (3)	1.0228 (3)	0.4546 (3)	0.0622 (12)	
H5A	0.5887	0.9811	0.4117	0.093*	
H5B	0.7061	0.9968	0.4473	0.093*	
O6	0.4400 (4)	0.8833 (4)	0.3526 (3)	0.0711 (13)	
H6A	0.3978	0.9092	0.3991	0.107*	
H6B	0.4082	0.9014	0.3094	0.107*	
C1	0.3993 (5)	0.4320 (5)	0.3376 (5)	0.0562 (17)	
H1	0.3943	0.4085	0.3905	0.067*	
C2	0.4103 (5)	0.5467 (6)	0.3634 (5)	0.0623 (18)	
H2	0.4143	0.6000	0.4323	0.075*	
C3	0.4153 (6)	0.5814 (5)	0.2856 (6)	0.0674 (19)	
H3	0.4206	0.6588	0.3004	0.081*	
C4	0.4124 (5)	0.4995 (6)	0.1847 (5)	0.0623 (18)	
H4	0.4159	0.5215	0.1309	0.075*	
C5	0.4043 (5)	0.3852 (5)	0.1639 (5)	0.0429 (14)	
C6	0.4060 (4)	0.2936 (5)	0.0575 (4)	0.0413 (14)	
C7	0.4251 (5)	0.3177 (6)	−0.0262 (5)	0.0568 (17)	
H7	0.4340	0.3933	−0.0172	0.068*	
C8	0.4309 (5)	0.2301 (6)	−0.1221 (5)	0.0570 (17)	
H8	0.4443	0.2457	−0.1784	0.068*	
C9	0.4166 (5)	0.1195 (6)	−0.1335 (5)	0.0501 (15)	
H9	0.4205	0.0587	−0.1976	0.060*	
C10	0.3961 (4)	0.0996 (5)	−0.0480 (5)	0.0464 (15)	
H10	0.3857	0.0243	−0.0562	0.056*	
C11	0.3500 (5)	0.2125 (5)	0.4401 (5)	0.0542 (16)	
H11	0.4272	0.1958	0.4352	0.065*	
C12	0.3092 (6)	0.2475 (6)	0.5350 (5)	0.0668 (19)	
H12	0.3571	0.2540	0.5930	0.080*	
C13	0.1967 (7)	0.2724 (6)	0.5417 (5)	0.070 (2)	
H13	0.1667	0.2984	0.6054	0.084*	
C14	0.1268 (6)	0.2589 (6)	0.4539 (5)	0.0622 (18)	
H14	0.0492	0.2747	0.4579	0.075*	
C15	0.1731 (5)	0.2216 (4)	0.3596 (4)	0.0415 (14)	
C16	0.1037 (5)	0.2016 (5)	0.2606 (4)	0.0435 (14)	
C17	−0.0148 (5)	0.2143 (6)	0.2558 (6)	0.0626 (18)	
H17	−0.0530	0.2360	0.3151	0.075*	
C18	−0.0748 (6)	0.1945 (7)	0.1626 (6)	0.083 (2)	
H18	−0.1545	0.2021	0.1582	0.099*	
C19	−0.0180 (6)	0.1636 (8)	0.0765 (6)	0.099 (3)	
H19	−0.0570	0.1515	0.0129	0.119*	
C20	0.1004 (5)	0.1506 (7)	0.0866 (5)	0.085 (3)	
H20	0.1396	0.1280	0.0278	0.101*	
C21	0.6248 (5)	0.2348 (5)	0.2767 (4)	0.0424 (14)	
C22	0.7075 (4)	0.2856 (5)	0.3740 (4)	0.0406 (14)	
C23	0.7796 (4)	0.3861 (5)	0.4152 (4)	0.0446 (14)	
H23	0.7892	0.4380	0.3864	0.054*	
C24	0.8374 (4)	0.3962 (5)	0.5108 (4)	0.0410 (14)	
C25	0.9183 (5)	0.4786 (5)	0.5900 (5)	0.0546 (16)	
H25	0.9451	0.5456	0.5856	0.066*	
C26	0.9571 (5)	0.4596 (6)	0.6732 (5)	0.0598 (18)	
H26	1.0104	0.5143	0.7256	0.072*	
C27	0.9183 (5)	0.3594 (6)	0.6811 (5)	0.0600 (18)	
H27	0.9473	0.3480	0.7381	0.072*	
C28	0.8382 (5)	0.2776 (5)	0.6066 (5)	0.0538 (16)	
H28	0.8114	0.2115	0.6126	0.065*	
C29	0.7985 (4)	0.2967 (5)	0.5217 (4)	0.0400 (13)	
C31	0.3117 (5)	−0.0931 (5)	0.1209 (5)	0.0449 (14)	
C32	0.2787 (4)	−0.2191 (5)	0.0709 (4)	0.0392 (13)	
C33	0.2308 (5)	−0.2888 (5)	0.1117 (5)	0.0524 (16)	
H33	0.2147	−0.2664	0.1799	0.063*	
C34	0.2103 (5)	−0.4011 (5)	0.0308 (4)	0.0480 (15)	
C35	0.1611 (5)	−0.5075 (5)	0.0239 (5)	0.0614 (18)	
H35	0.1328	−0.5130	0.0810	0.074*	
C36	0.1561 (6)	−0.6015 (6)	−0.0683 (6)	0.074 (2)	
H36	0.1226	−0.6717	−0.0742	0.088*	
C37	0.1997 (6)	−0.5951 (6)	−0.1537 (6)	0.070 (2)	
H37	0.1962	−0.6615	−0.2151	0.084*	
C38	0.2484 (6)	−0.4927 (6)	−0.1504 (5)	0.0606 (17)	
H38	0.2776	−0.4890	−0.2079	0.073*	
C39	0.2515 (5)	−0.3961 (5)	−0.0576 (4)	0.0441 (14)	
C41	−0.1487 (6)	0.9382 (7)	0.3045 (6)	0.083 (2)	
H41	−0.2270	0.9264	0.2776	0.100*	
C42	−0.0659 (7)	0.9251 (6)	0.2373 (6)	0.077 (2)	
H42	−0.0875	0.9037	0.1671	0.092*	
C43	0.0489 (6)	0.9446 (5)	0.2767 (5)	0.0643 (18)	
H43	0.1077	0.9376	0.2339	0.077*	
C44	0.0766 (5)	0.9749 (5)	0.3810 (5)	0.0550 (16)	
H44	0.1547	0.9888	0.4090	0.066*	
C45	−0.0117 (5)	0.9847 (5)	0.4443 (4)	0.0465 (15)	

### Atomic displacement parameters (Å^2^)

**Table:**

	*U*^11^	*U*^22^	*U*^33^	*U*^12^	*U*^13^	*U*^23^
Cd1	0.0375 (2)	0.0444 (3)	0.0352 (2)	0.00611 (17)	0.00440 (16)	0.01586 (19)
N1	0.049 (3)	0.040 (3)	0.050 (3)	0.009 (2)	0.009 (2)	0.019 (3)
N2	0.042 (3)	0.044 (3)	0.036 (3)	0.009 (2)	0.005 (2)	0.015 (2)
N3	0.051 (3)	0.045 (3)	0.027 (3)	0.007 (2)	0.008 (2)	0.011 (2)
N4	0.035 (3)	0.075 (4)	0.043 (3)	0.014 (2)	0.006 (2)	0.023 (3)
N5	0.048 (3)	0.042 (3)	0.037 (3)	0.000 (2)	−0.002 (2)	0.018 (2)
N6	0.053 (3)	0.051 (3)	0.034 (3)	0.006 (2)	0.005 (2)	0.019 (3)
N7	0.047 (3)	0.085 (4)	0.063 (4)	0.000 (3)	−0.002 (3)	0.031 (3)
O1	0.042 (2)	0.050 (3)	0.056 (3)	0.0005 (19)	−0.0068 (19)	0.022 (2)
O2	0.048 (2)	0.101 (4)	0.048 (3)	0.000 (2)	0.006 (2)	0.044 (3)
O3	0.071 (3)	0.044 (3)	0.050 (3)	0.001 (2)	0.010 (2)	0.011 (2)
O4	0.063 (3)	0.047 (3)	0.070 (3)	0.007 (2)	0.010 (2)	0.036 (2)
O5	0.051 (2)	0.071 (3)	0.068 (3)	0.002 (2)	0.001 (2)	0.035 (3)
O6	0.083 (3)	0.072 (3)	0.054 (3)	−0.004 (3)	0.005 (2)	0.026 (3)
C1	0.069 (4)	0.047 (4)	0.044 (4)	0.008 (3)	0.019 (3)	0.009 (3)
C2	0.062 (4)	0.048 (4)	0.061 (5)	0.009 (3)	0.017 (3)	0.007 (4)
C3	0.074 (5)	0.033 (4)	0.088 (6)	0.008 (3)	0.013 (4)	0.018 (4)
C4	0.068 (4)	0.053 (4)	0.065 (5)	0.010 (3)	0.011 (3)	0.024 (4)
C5	0.039 (3)	0.037 (4)	0.055 (4)	0.010 (3)	0.003 (3)	0.022 (3)
C6	0.035 (3)	0.049 (4)	0.046 (4)	0.007 (3)	0.005 (3)	0.026 (3)
C7	0.064 (4)	0.056 (4)	0.061 (4)	0.007 (3)	0.009 (3)	0.035 (4)
C8	0.071 (4)	0.069 (5)	0.039 (4)	0.014 (4)	0.012 (3)	0.029 (4)
C9	0.045 (3)	0.063 (4)	0.042 (4)	0.018 (3)	0.008 (3)	0.021 (3)
C10	0.042 (3)	0.054 (4)	0.049 (4)	0.008 (3)	0.005 (3)	0.027 (3)
C11	0.059 (4)	0.062 (4)	0.040 (4)	0.007 (3)	0.009 (3)	0.020 (3)
C12	0.084 (5)	0.074 (5)	0.043 (4)	0.004 (4)	0.006 (4)	0.027 (4)
C13	0.098 (6)	0.070 (5)	0.046 (4)	0.023 (4)	0.029 (4)	0.023 (4)
C14	0.061 (4)	0.071 (5)	0.056 (4)	0.023 (4)	0.026 (3)	0.023 (4)
C15	0.044 (3)	0.034 (3)	0.046 (4)	0.007 (3)	0.017 (3)	0.014 (3)
C16	0.040 (3)	0.038 (3)	0.049 (4)	0.007 (3)	0.010 (3)	0.014 (3)
C17	0.039 (3)	0.076 (5)	0.076 (5)	0.016 (3)	0.018 (3)	0.031 (4)
C18	0.045 (4)	0.120 (7)	0.092 (6)	0.020 (4)	0.006 (4)	0.054 (6)
C19	0.052 (5)	0.179 (9)	0.074 (6)	0.018 (5)	−0.008 (4)	0.064 (6)
C20	0.046 (4)	0.156 (8)	0.053 (5)	0.018 (4)	0.001 (3)	0.045 (5)
C21	0.036 (3)	0.048 (4)	0.046 (4)	0.019 (3)	0.007 (3)	0.020 (3)
C22	0.033 (3)	0.055 (4)	0.035 (3)	0.009 (3)	0.007 (2)	0.019 (3)
C23	0.039 (3)	0.049 (4)	0.049 (4)	0.000 (3)	0.007 (3)	0.025 (3)
C24	0.035 (3)	0.044 (4)	0.043 (4)	0.003 (3)	0.006 (3)	0.018 (3)
C25	0.056 (4)	0.050 (4)	0.053 (4)	−0.007 (3)	0.001 (3)	0.020 (3)
C26	0.057 (4)	0.063 (5)	0.046 (4)	−0.012 (3)	−0.004 (3)	0.015 (4)
C27	0.050 (4)	0.076 (5)	0.053 (4)	−0.001 (3)	−0.007 (3)	0.030 (4)
C28	0.055 (4)	0.059 (4)	0.053 (4)	−0.002 (3)	−0.002 (3)	0.032 (4)
C29	0.037 (3)	0.047 (4)	0.037 (3)	0.002 (3)	0.004 (2)	0.020 (3)
C31	0.034 (3)	0.043 (4)	0.054 (4)	0.011 (3)	0.008 (3)	0.015 (3)
C32	0.042 (3)	0.037 (3)	0.037 (3)	0.008 (3)	0.008 (2)	0.013 (3)
C33	0.063 (4)	0.053 (4)	0.043 (4)	0.002 (3)	0.013 (3)	0.021 (3)
C34	0.048 (3)	0.046 (4)	0.044 (4)	0.004 (3)	0.003 (3)	0.015 (3)
C35	0.071 (4)	0.048 (4)	0.066 (5)	−0.004 (3)	0.009 (3)	0.027 (4)
C36	0.086 (5)	0.047 (5)	0.080 (6)	−0.005 (4)	−0.010 (4)	0.023 (4)
C37	0.094 (5)	0.043 (4)	0.057 (5)	0.008 (4)	−0.013 (4)	0.008 (4)
C38	0.075 (5)	0.062 (5)	0.039 (4)	0.013 (4)	−0.003 (3)	0.016 (4)
C39	0.046 (3)	0.045 (4)	0.036 (3)	0.003 (3)	−0.002 (3)	0.013 (3)
C41	0.058 (5)	0.114 (7)	0.067 (5)	−0.005 (4)	−0.007 (4)	0.033 (5)
C42	0.088 (6)	0.080 (6)	0.053 (5)	0.002 (4)	−0.001 (4)	0.022 (4)
C43	0.079 (5)	0.055 (4)	0.061 (5)	0.014 (4)	0.017 (4)	0.024 (4)
C44	0.048 (4)	0.052 (4)	0.070 (5)	0.007 (3)	0.007 (3)	0.030 (4)
C45	0.045 (3)	0.041 (4)	0.054 (4)	0.001 (3)	0.002 (3)	0.022 (3)

### Geometric parameters (Å, °)

**Table:**

Cd1—O1	2.239 (4)	C12—H12	0.9300
Cd1—O4	2.336 (4)	C13—C14	1.378 (9)
Cd1—N2	2.364 (4)	C13—H13	0.9300
Cd1—N3	2.408 (4)	C14—C15	1.389 (7)
Cd1—N4	2.419 (4)	C14—H14	0.9300
Cd1—N1	2.450 (5)	C15—C16	1.484 (8)
Cd1—O3	2.653 (4)	C16—C17	1.385 (7)
N1—C1	1.333 (7)	C17—C18	1.368 (9)
N1—C5	1.340 (7)	C17—H17	0.9300
N2—C10	1.344 (7)	C18—C19	1.357 (9)
N2—C6	1.347 (7)	C18—H18	0.9300
N3—C11	1.333 (7)	C19—C20	1.388 (9)
N3—C15	1.341 (6)	C19—H19	0.9300
N4—C20	1.325 (7)	C20—H20	0.9300
N4—C16	1.335 (7)	C21—C22	1.491 (7)
N5—C22	1.371 (6)	C22—C23	1.365 (7)
N5—C29	1.372 (6)	C23—C24	1.425 (7)
N5—H5C	0.8600	C23—H23	0.9300
N6—C32	1.370 (6)	C24—C25	1.407 (7)
N6—C39	1.375 (7)	C24—C29	1.410 (7)
N6—H6C	0.8600	C25—C26	1.364 (8)
N7—C41	1.336 (8)	C25—H25	0.9300
N7—C45	1.341 (7)	C26—C27	1.395 (8)
O1—C21	1.290 (6)	C26—H26	0.9300
O2—C21	1.236 (6)	C27—C28	1.371 (8)
O3—C31	1.261 (7)	C27—H27	0.9300
O4—C31	1.250 (7)	C28—C29	1.389 (7)
O5—H5A	0.8200	C28—H28	0.9300
O5—H5B	0.8200	C31—C32	1.486 (7)
O6—H6A	0.8200	C32—C33	1.372 (7)
O6—H6B	0.8200	C33—C34	1.415 (8)
C1—C2	1.364 (8)	C33—H33	0.9300
C1—H1	0.9300	C34—C39	1.408 (7)
C2—C3	1.368 (9)	C34—C35	1.413 (8)
C2—H2	0.9300	C35—C36	1.359 (9)
C3—C4	1.380 (8)	C35—H35	0.9300
C3—H3	0.9300	C36—C37	1.387 (9)
C4—C5	1.376 (8)	C36—H36	0.9300
C4—H4	0.9300	C37—C38	1.384 (9)
C5—C6	1.486 (8)	C37—H37	0.9300
C6—C7	1.387 (7)	C38—C39	1.383 (8)
C7—C8	1.373 (8)	C38—H38	0.9300
C7—H7	0.9300	C41—C42	1.371 (9)
C8—C9	1.368 (8)	C41—H41	0.9300
C8—H8	0.9300	C42—C43	1.361 (9)
C9—C10	1.384 (7)	C42—H42	0.9300
C9—H9	0.9300	C43—C44	1.377 (8)
C10—H10	0.9300	C43—H43	0.9300
C11—C12	1.369 (8)	C44—C45	1.389 (8)
C11—H11	0.9300	C44—H44	0.9300
C12—C13	1.356 (9)	C45—C45^i^	1.468 (11)
			
O1—Cd1—O4	107.11 (14)	N3—C15—C14	120.3 (5)
O1—Cd1—N2	108.36 (14)	N3—C15—C16	116.3 (5)
O4—Cd1—N2	82.76 (15)	C14—C15—C16	123.4 (5)
O1—Cd1—N3	89.13 (15)	N4—C16—C17	121.4 (6)
O4—Cd1—N3	119.52 (14)	N4—C16—C15	117.2 (5)
N2—Cd1—N3	146.91 (16)	C17—C16—C15	121.4 (5)
O1—Cd1—N4	156.39 (15)	C18—C17—C16	119.2 (6)
O4—Cd1—N4	89.06 (16)	C18—C17—H17	120.4
N2—Cd1—N4	90.32 (16)	C16—C17—H17	120.4
N3—Cd1—N4	67.69 (16)	C19—C18—C17	119.9 (6)
O1—Cd1—N1	89.04 (15)	C19—C18—H18	120.0
O4—Cd1—N1	150.16 (15)	C17—C18—H18	120.0
N2—Cd1—N1	68.19 (16)	C18—C19—C20	117.8 (7)
N3—Cd1—N1	84.76 (16)	C18—C19—H19	121.1
N4—Cd1—N1	84.58 (16)	C20—C19—H19	121.1
O1—Cd1—O3	84.88 (14)	N4—C20—C19	123.2 (6)
O4—Cd1—O3	52.23 (13)	N4—C20—H20	118.4
N2—Cd1—O3	134.85 (15)	C19—C20—H20	118.4
N3—Cd1—O3	72.87 (14)	O2—C21—O1	125.9 (5)
N4—Cd1—O3	92.11 (15)	O2—C21—C22	119.4 (5)
N1—Cd1—O3	156.86 (15)	O1—C21—C22	114.6 (5)
C1—N1—C5	118.9 (5)	C23—C22—N5	109.8 (5)
C1—N1—Cd1	123.7 (4)	C23—C22—C21	129.4 (5)
C5—N1—Cd1	117.4 (4)	N5—C22—C21	120.8 (5)
C10—N2—C6	118.2 (5)	C22—C23—C24	106.9 (5)
C10—N2—Cd1	121.1 (4)	C22—C23—H23	126.5
C6—N2—Cd1	120.6 (4)	C24—C23—H23	126.5
C11—N3—C15	118.6 (5)	C25—C24—C29	118.0 (5)
C11—N3—Cd1	121.7 (4)	C25—C24—C23	135.2 (5)
C15—N3—Cd1	119.7 (4)	C29—C24—C23	106.8 (5)
C20—N4—C16	118.5 (5)	C26—C25—C24	119.6 (6)
C20—N4—Cd1	122.5 (4)	C26—C25—H25	120.2
C16—N4—Cd1	119.1 (4)	C24—C25—H25	120.2
C22—N5—C29	108.8 (4)	C25—C26—C27	121.1 (6)
C22—N5—H5C	125.6	C25—C26—H26	119.4
C29—N5—H5C	125.6	C27—C26—H26	119.4
C32—N6—C39	109.0 (5)	C28—C27—C26	121.3 (6)
C32—N6—H6C	125.5	C28—C27—H27	119.3
C39—N6—H6C	125.5	C26—C27—H27	119.3
C41—N7—C45	117.4 (6)	C27—C28—C29	117.7 (6)
C21—O1—Cd1	119.1 (3)	C27—C28—H28	121.1
C31—O3—Cd1	84.5 (3)	C29—C28—H28	121.1
C31—O4—Cd1	99.5 (4)	N5—C29—C28	130.1 (5)
H5A—O5—H5B	114.5	N5—C29—C24	107.7 (5)
H6A—O6—H6B	100.4	C28—C29—C24	122.3 (5)
N1—C1—C2	123.1 (6)	O4—C31—O3	123.7 (6)
N1—C1—H1	118.4	O4—C31—C32	118.5 (5)
C2—C1—H1	118.4	O3—C31—C32	117.7 (5)
C1—C2—C3	118.5 (6)	N6—C32—C33	109.2 (5)
C1—C2—H2	120.7	N6—C32—C31	121.3 (5)
C3—C2—H2	120.7	C33—C32—C31	129.6 (5)
C2—C3—C4	118.9 (6)	C32—C33—C34	107.4 (5)
C2—C3—H3	120.6	C32—C33—H33	126.3
C4—C3—H3	120.6	C34—C33—H33	126.3
C5—C4—C3	119.9 (6)	C39—C34—C35	119.1 (6)
C5—C4—H4	120.0	C39—C34—C33	106.9 (5)
C3—C4—H4	120.0	C35—C34—C33	134.1 (6)
N1—C5—C4	120.6 (6)	C36—C35—C34	118.5 (6)
N1—C5—C6	117.1 (5)	C36—C35—H35	120.8
C4—C5—C6	122.3 (6)	C34—C35—H35	120.8
N2—C6—C7	121.1 (5)	C35—C36—C37	121.5 (6)
N2—C6—C5	116.5 (5)	C35—C36—H36	119.3
C7—C6—C5	122.3 (5)	C37—C36—H36	119.3
C8—C7—C6	120.1 (6)	C38—C37—C36	122.0 (6)
C8—C7—H7	119.9	C38—C37—H37	119.0
C6—C7—H7	119.9	C36—C37—H37	119.0
C9—C8—C7	118.9 (6)	C39—C38—C37	117.0 (6)
C9—C8—H8	120.6	C39—C38—H38	121.5
C7—C8—H8	120.6	C37—C38—H38	121.5
C8—C9—C10	119.0 (6)	N6—C39—C38	130.4 (6)
C8—C9—H9	120.5	N6—C39—C34	107.6 (5)
C10—C9—H9	120.5	C38—C39—C34	122.0 (6)
N2—C10—C9	122.7 (6)	N7—C41—C42	124.7 (7)
N2—C10—H10	118.7	N7—C41—H41	117.6
C9—C10—H10	118.7	C42—C41—H41	117.6
N3—C11—C12	123.9 (6)	C43—C42—C41	117.8 (7)
N3—C11—H11	118.1	C43—C42—H42	121.1
C12—C11—H11	118.1	C41—C42—H42	121.1
C13—C12—C11	117.8 (6)	C42—C43—C44	119.0 (6)
C13—C12—H12	121.1	C42—C43—H43	120.5
C11—C12—H12	121.1	C44—C43—H43	120.5
C12—C13—C14	119.9 (6)	C43—C44—C45	120.2 (6)
C12—C13—H13	120.1	C43—C44—H44	119.9
C14—C13—H13	120.1	C45—C44—H44	119.9
C13—C14—C15	119.5 (6)	N7—C45—C44	120.8 (6)
C13—C14—H14	120.2	N7—C45—C45^i^	116.2 (6)
C15—C14—H14	120.2	C44—C45—C45^i^	122.9 (7)

Symmetry codes: (i) −*x*, −*y*+2, −*z*+1.

### Hydrogen-bond geometry (Å, °)

**Table:**

*D*—H···*A*	*D*—H	H···*A*	*D*···*A*	*D*—H···*A*
N5—H5C···O5^ii^	0.86	2.09	2.892 (9)	155
N6—H6C···O2^iii^	0.86	2.00	2.801 (8)	156
O5—H5A···O6	0.82	1.96	2.748 (8)	161
O5—H5B···N7^iv^	0.82	2.09	2.879 (8)	161
O6—H6A···O5^v^	0.82	2.01	2.781 (7)	156
O6—H6B···O3^vi^	0.82	1.95	2.778 (6)	174

Symmetry codes: (ii) *x*, *y*−1, *z*; (iii) −*x*+1, −*y*, −*z*;  (iv) *x*+1, *y*, *z*; (v) −*x*+1, −*y*+2, −*z*+1; (vi) *x*, *y*+1, *z*.
